# Venous thromboembolism and intracranial hemorrhage after craniotomy for primary malignant brain tumors: a National Surgical Quality Improvement Program analysis

**DOI:** 10.1007/s11060-017-2631-5

**Published:** 2017-10-16

**Authors:** Joeky T. Senders, Nicole H. Goldhaber, David J. Cote, Ivo S. Muskens, Hassan Y. Dawood, Filip Y. F. L. De Vos, William B. Gormley, Timothy R. Smith, Marike L. D. Broekman

**Affiliations:** 1 Computational Neurosciences Outcomes Center, Department of Neurosurgery, Brigham and Women’s Hospital, Harvard Medical School, 60 Fenwood Road, Boston, MA 02115 USA; 2 Department of Neurosurgery, University Medical Center Utrecht, Heidelberglaan 100, 3584 CX Utrecht, The Netherlands; 3 Department of Medical Oncology, University Medical Center Utrecht, Heidelberglaan 100, 3584 CX Utrecht, The Netherlands

**Keywords:** Deep venous thrombosis, Intracranial hemorrhage, Primary malignant brain tumor, Pulmonary embolism, Venous thromboembolism

## Abstract

Venous thromboembolism (VTE), including deep venous thrombosis (DVT) and pulmonary embolism (PE), frequently complicates the postoperative course of primary malignant brain tumor patients. Thromboprophylactic anticoagulation is commonly used to prevent VTE at the risk of intracranial hemorrhage (ICH). We extracted all patients who underwent craniotomy for a primary malignant brain tumor from the National Surgical Quality Improvement Program (NSQIP) registry (2005–2015) to perform a time-to-event analysis and identify relevant predictors of DVT, PE, and ICH within 30 days after surgery. Among the 7376 identified patients, the complication rates were 2.6, 1.5, and 1.3% for DVT, PE, and ICH, respectively. VTE was the second-most common major complication and third-most common reason for readmission. ICH was the most common reason for reoperation. The increased risk of VTE extends beyond the period of hospitalization, especially for PE, whereas ICH occurred predominantly within the first days after surgery. Older age and higher BMI were overall predictors of VTE. Dependent functional status and longer operative times were predictive for VTE during hospitalization, but not for post-discharge events. Admission two or more days before surgery was predictive for DVT, but not for PE. Preoperative steroid usage and male gender were predictive for post-discharge DVT and PE, respectively. ICH was associated with various comorbidities and longer operative times. This multicenter study demonstrates distinct critical time periods for the development of thrombotic and hemorrhagic events after craniotomy. Furthermore, the VTE risk profile depends on the type of VTE (DVT vs. PE) and clinical setting (hospitalized vs. post-discharge patients).

## Introduction

Venous thromboembolism (VTE), including deep venous thrombosis (DVT) and pulmonary embolism (PE), is a major cause of morbidity and mortality in patients undergoing craniotomy for primary malignant brain tumors [1–6]. In addition to known surgical risk factors, such as venous stasis from perioperative immobility, endothelial injury, and inflammation from the operation itself, cancer is a recognized risk factor for VTE development [7]. Among all cancer types, high-grade gliomas have been shown to result in second highest lifetime risk for cancer-related VTE, one of the highest risks of perioperative VTE and, when comparing craniotomy for any brain tumor to craniotomy for non-neoplastic disease, rates of postoperative VTE have been reported to be twice as high [8–11]. VTE has been reported as one of the most frequent major complications after craniotomy for brain tumors with incidences up to 21% in the first 3 months after surgery [1–6].

Previous studies have identified older age, male sex, Hispanic ethnicity, history of craniotomy, history of VTE, congestive heart failure, coagulopathy, seizures, increased stay on the intensive care unit, prolonged hospital stay, medium bed size, residual tumor tissue, and absence of thromboprophylactic therapy as predictors of VTE after craniotomy for primary malignant brain tumors [3, 4, 6, 12–15]. Most of these studies have been small, single-center studies, and none of these studies have identified predictors or performed time-to-event analyses separated for VTE type (DVT vs. PE) and the clinical setting (hospitalized vs. post-discharge patients).

Most patients undergoing surgery for brain tumors receive pharmaceutic prophylaxis in combination with mechanical prophylaxis in the perioperative setting [16–19]. However, anticoagulation increases the risk of intracranial hemorrhage (ICH), which is one of the most frequent and feared complications in patients undergoing operations for brain tumors [20]. The increased risk of ICH makes the use of prophylactic anticoagulation an issue of great debate and careful balance in this patient population. Although the incidence of ICH is lower compared to VTE events, their outcomes can be at least as detrimental. Only few predictors associated with ICH have been identified including history of craniotomy, use of bevacizumab, and therapeutic anticoagulation for a VTE [13, 20–24]. Adequate assessment of the perioperative risk of both VTE and ICH among this patient population, as well as accurately predicting the typical time to a thrombotic or hemorrhagic event, is meaningful in tailoring postoperative management to the risk profile of the individual patient.

The American College of Surgeons (ACS) National Surgical Quality Improvement Program (NSQIP) database registers follow-up of neurosurgical patients for 30 days postoperatively, and reports variables relevant to this issue, including the occurrence of DVT, PE, and ICH with related time-to-event data. Because the risk of ICH is intimately tied to the thromboprophylactic treatment used for patients with brain tumors, this study addresses thrombotic as well as hemorrhagic complications. In this study, NSQIP was used to identify predictors and perform time-to-event analyses for VTE and ICH. Assessment of VTE was stratified for VTE type (DVT vs. PE) and clinical setting (hospitalized vs. post-discharge patients).

## Methods

### Data source

The NSQIP includes surgical patients from over 600 participating hospitals in the U.S. operated on from 2005 to 2015. This validated dataset is collected by trained surgical reviewers using a standardized protocol, and includes common postoperative complications, occurrence of reoperations and readmissions, together with associated reasons and time-to-event in days. The NSQIP registry has previously been used to study outcomes after neurosurgical procedures [25–36]. Our institutional review board has exempted the NSQIP database from review.

### Inclusion criteria

Patients were included who met the following criteria: (1) age 18 years or older; (2) a Current Procedural Terminology (CPT) code indicating craniotomy for surgical resection of brain tumor (CPT: 61500, 61510, 61512, 61518, 61519, 61520, 61521, 61526, and 61530); (3) a postoperative diagnosis indicative of primary malignant brain tumor (International Classification of Diseases, Ninth Revision [ICD-9]: 191.x).

### Covariates

Age, body mass index (BMI), and operative time were assessed continuously in years, kg/m^2^, and minutes, respectively. Other categorized pre- and perioperative covariates included sex, race, American Society of Anesthesiologists (ASA)-classification (I–II, III or IV–V), functional status (dependent or independent), smoking within 1 year prior to surgery, history of hypertension requiring medication, chronic heart failure, COPD, renal failure, dialysis, insulin dependent diabetes, bleeding disorder, weight loss (> 10% loss of body weight in the 6 months prior to surgery), dyspnea, ventilator dependence, steroid usage, emergency classification, transfer status (admitted from home vs. not from home), creatinine (< 1.4 vs. ≥ 1.4 mg/dL), hematocrit (< 36 vs. ≥36%), platelet count (100–450/µm^3^, < 100/µm^3^, vs. > 450/µm^3^), sodium (135–145 mEq/L, < 135 mEq/L, vs. > 145 mEq/L), white blood cell (WBC) count (≤ 12,000/µL vs. > 12,000/µL), preoperative transfusion, preoperative systemic inflammatory response syndrome, admission two or more days before surgery, and anesthesia type (general vs. no general anesthesia).

### Missing data

Covariates with more than 10% missing data or occurring in less than 1% of cases were excluded from multivariable analysis. Cases with missing data in one of the variables of the multivariable analysis were excluded from the analysis. A confirmatory analysis was performed for every multivariable model, in which missing data was coded as an additional group to verify if missing data affected the results.

### Outcomes

VTE was defined as the occurrence of DVT or PE within 30 days after surgery. The occurrence of other major complications and reasons for readmission and reoperation were extracted by means of ICD-9 and CPT codes, to assess the relative contribution of VTE and ICH to morbidity, readmission, and reoperation. Based on a previously published definition, major complications were defined as either acute renal failure, cardiac arrest, death, failure to wean from ventilator, myocardial infarction, reintubation, reoperation, stroke, VTE, sepsis, and surgical site infection [37]. ICH was defined as the occurrence of an ICH requiring surgical evacuation and extracted by means of CPT and ICD-9 codes. Reasons for reoperation including ICH were collected since 2012.

### Statistical analysis

Statistical analyses were conducted using R 3.3.3 (R Core Team, Auckland, New Zealand). Univariable analysis was performed using logistic regression. Each primary thrombotic outcome (VTE, DVT, PE) was assessed for its occurrence during the initial hospital stay, after discharge, and within 30 days overall. ICH was assessed within 30 days overall. Potential predictors were selected for inclusion in the multivariable logistic regression analysis based on univariable analysis for each outcome. Only pre- and intraoperative factors were included in the multivariable analysis, because inclusion of postoperative complications other than VTE or ICH would reduce the timeframe in which complications can be detected due to the limited 30-day collection timeframe of NSQIP, thereby biasing the results of the model. Potential predictors were excluded from the final model if they demonstrated multicollinearity or had a relative low contribution to model fit. A p value below 0.05 was considered statistically significant. Bonferroni correction for multiple testing was deemed to be too rigorous due to the low number of events. The β-coefficients of the continuous variables in the final model were multiplied to represent the odds ratios and confidence intervals of meaningful and interpretable units for age (per 10 years increase), BMI (per 5 kg/m^2^ increase), and operative time (per 60 min increase).

## Results

### Demographics of study population

The NSQIP registry provided 7376 patients who underwent craniotomy for resection of a primary malignant brain tumor during the study period. Comorbidities, demographics, and preoperative laboratory values are shown separated by the occurrence of VTE (Table 1).

**Table 1:** Demographics and preoperative comorbidities of NSQIP patients undergoing craniotomy for glioma, compared by VTE occurrence

Characteristic (%)	Definition	Total (n = 7376)	No VTE (n = 7119)	VTE (n = 257)	Odd ratio	95% CI	p value
Age	Years ± SD	54.5 ± 15.6	54.4 ± 15.6	59.4 ± 15.7	1.25^a^	1.15–1.36	< **0.001**
Gender	Female	42.3	42.3	41.6	Ref	–	–
	Male	57.7	57.7	58.4	1.03	0.91–1.19	0.82
Race	White	91.7	91.7	94.8	Ref	–	–
	Black	4.8	4.8	4.2	0.85	0.40–1.60	0.66
	Asian	3.0	3.0	0.5	0.15	0.01–0.67	**0.04**
	Other	0.5	0.5	0.5	0.95	0.05–4.51	0.96
ASA Classification	I–II	27.7	28.1	18.3	Ref	–	–
	III	59.2	59.0	65.5	1.71	1.23–2.40	**0.002**
	IV–V	13.0	12.9	16.3	1.94	1.26–2.97	**0.002**
BMI	kg/m^2^ ± SD	28.4 ± 6.2	28.4 ± 6.2	29.8 ± 6.3	1.19^b^	1.08–1.30	< **0.001**
Smoking		17.1	17.4	10.1	0.53	0.35–0.78	**0.003**
Emergency case		6.5	6.5	8.9	1.42	0.89–2.15	0.12
Admitted not from home		18.1	18.4	24.2	1.42	1.05–1.89	**0.02**
Hypertension		35.4	35.1	43.6	1.43	1.11–1.83	**0.006**
History of COPD		2.4	2.3	3.1	1.35	0.60–2.59	0.42
History of CHF		0.2	0.2	0.8	3.48	0.55–12.32	0.10
Renal Failure		0.1	0.1	0.4	4.63	0.24–27.24	0.16
Dialysis		0.1	0.1	0.0	Inf.^d^	Inf.^d^	1.000
Ventilator dependence		1.1	1.1	3.1	2.98	1.31–5.86	**0.004**
Weight loss		1.7	1.7	1.6	0.93	0.28–2.23	0.89
Bleeding disorder		2.2	2.1	3.1	1.46	0.65–2.82	0.30
Dyspnea		2.6	2.6	2.7	1.07	0.45–2.12	0.87
Insulin-dependent diabetes		3.9	3.8	4.7	1.23	0.65–2.13	0.49
Preoperative steroid usage		16.6	16.4	23.3	1.55	1.15–2.07	**0.004**
Dependent functional status		5.1	5.0	10.4	2.23	1.43–3.32	< **0.001**
Preoperative SIRS		3.6	3.4	5.1	1.51	0.81–2.56	0.16
Preoperative transfusion		0.2	0.2	0.4	1.85	0.10–9.18	0.55
Preoperative Sodium	135–145	89.9	90.0	87.2	Ref	–	–
	< 135	9.1	9.0	10.8	1.24	0.80–1.83	0.31
	> 145	1.0	1.0	2.0	2.07	0.72–4.69	0.12
Preoperative creatinine	≥ 1.4 mg/dL	4.4	4.6	4.4	0.96	0.49–1.69	0.90
Preoperative WBC	> 12,000/µL	24.8	24.4	33.1	1.53	1.16–1.99	**0.002**
Preoperative Hematocrit	< 36%	11.9	11.8	12.6	1.07	0.72–1.54	0.71
Platelets	100–450	97.5	97.6	95.7	Ref	–	–
	< 100	1.3	1.0	2.8	2.29	0.95–4.65	0.05
	> 450	1.2	1.2	1.6	1.37	0.41–3.33	0.54
Operative time	Minutes [IQR]	179 [123–250]	179 [123–250]	191 [134–252]	1.33^c^	1.02–1.74	**0.04**
No general anesthesia		5.9	5.9	5.4	0.91	0.50–1.52	0.74
Admission to operation	≥ 2 days	32.8	32.4	46.3	1.80	1.40–2.31	< **0.001**

Bold p-values below 0.05 were considered as statistically significant

*ASA* American Society of Anesthesiologists, *CI* confidence interval, *CHF* congestive heart failure, *SIRS* systematic inflammatory response syndrome, *WBC* white blood cell count

^a^Inflated β-coefficients to odds ratio per 10 years increase

^b^Inflated β-coefficients to odds ratio per 5 kg/m^2^ increase

^c^Inflated β-coefficients to odds ratio per 60 min increase

^d^Infinity due to 0 count in one of the cells

### Outcomes

**Fig. 1:**
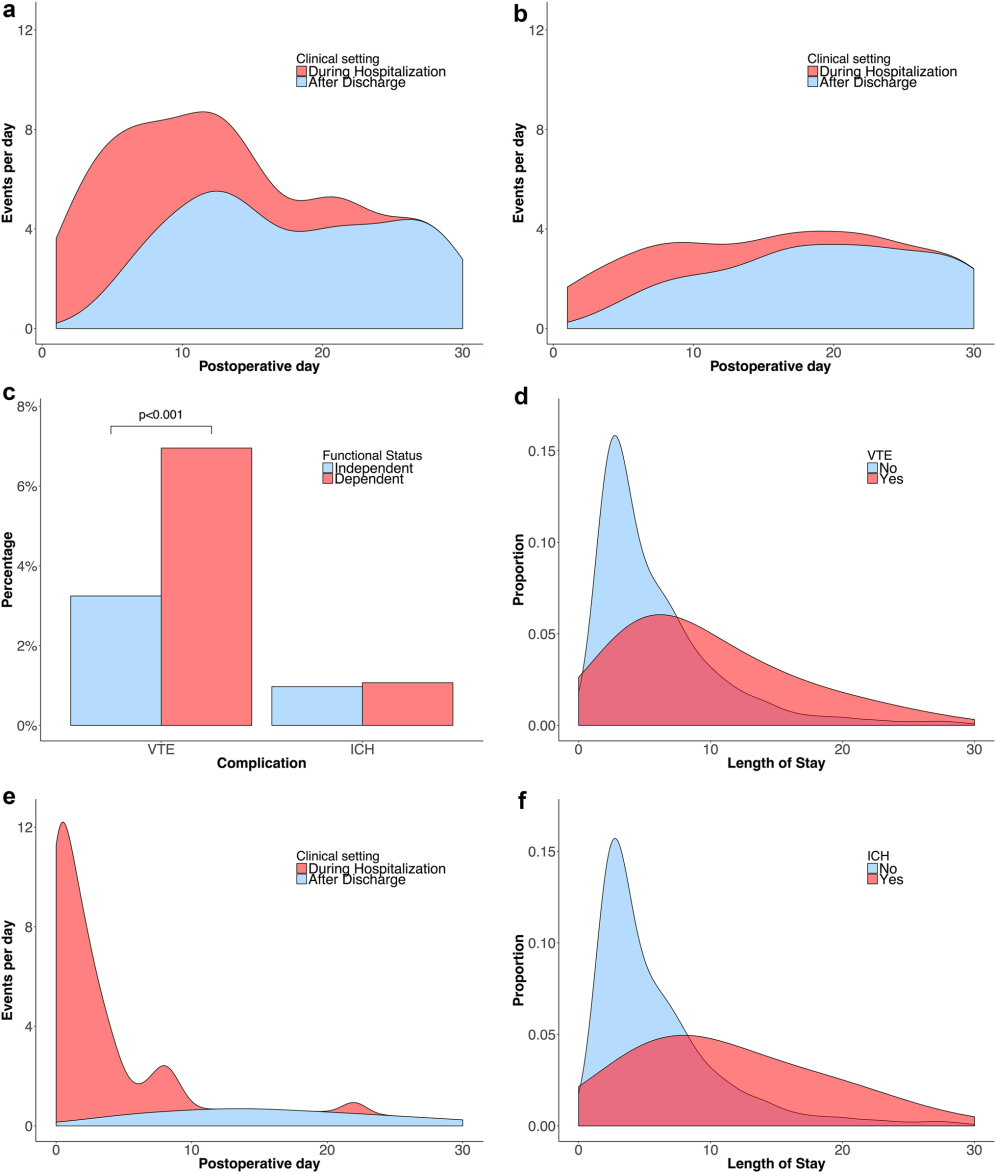
Number of patients per day in the total population developing a deep venous thrombosis (**a**), pulmonary embolism (**b**) or intracranial hemorrhage (**e**) after craniotomy stratified for timing of diagnosis. Distribution of length of postoperative stay compared by the occurrence of VTE (**d**) and ICH (**f**). Frequency of VTE and ICH compared by functional status (**c**)

Of the 7376 patients that were identified, 257 (3.5%) developed a VTE within 30 days after surgery, of which 91 (36%) occurred within the initial hospital stay. VTE was the second-most common major complication and included 192 DVTs (2.6%) and 107 PEs (1.5%). Forty-two patients developed both DVT and PE (0.6%). The rate of DVT was highest within the first 2 weeks after surgery, whereas the rate of PE was fairly consistent throughout the first month, occurring predominantly post-discharge (Fig. 1a, b). The rate of VTE was more than twice as high (7.0 vs. 3.2%, p < 0.001) among patients with a preoperative dependent functional status compared to patients with an independent functional status (Fig. 1c).

The median length of stay among VTE patients was 8 days (inter-quartile range [IQR] 5–16 days) compared to 4 days (IQR 3–8 days) in non-VTE patients (Fig. 1d). In-hospital VTE occurred at a median of 6 days (IQR 3–8 days) after surgery, and patients were discharged at a median of 8 days after the occurrence of VTE (IQR 3–16 days). Post-discharge VTE occurred at a median of 13 days after discharge (IQR 6–19 days) and resulted in 25% of cases in readmission, making VTE the third-most common reason for readmission (7.4% of total readmissions). Of the patients that developed a PE, 35 (32.7%) were preceded by a DVT. Post-discharge PEs were less frequently preceded by a DVT than hospital acquired PEs (26 vs. 48%, p = 0.048).

Of the 5699 patients that were identified in the NSQIP registry 2012–2015 with data on reoperation and associated reasons, 72 (1.3%) developed an ICH requiring surgical evacuation at a median of 2 days after surgery (IQR 0–7.5 days) (Fig. 1e). ICH was the most common reason for reoperation (18.5% of the total number of reoperations). The median length of stay among ICH patients was 12 days (IQR 6–21 days) compared to 4 days (IQR 3–8 days) in non-ICH patients (Fig. 1f). The 55 patients (77.5%) that developed an ICH during the initial hospital stay, were discharged at a median of 10 days (IQR 6–19) after the occurrence of ICH.

### Multivariable analysis

Older age and higher BMI were found to be risk factors of VTE overall (Tables 2, 3). Dependent functional status and longer operative times were predictive for hospital VTE, but not for post-discharge events. Admission two or more days before surgery was a predictor of DVT, but not for PE. Steroid usage was predictive for post-discharge DVT, and male gender was predictive for post-discharge PE. Higher ASA-classification, hypertension, weight loss, bleeding disorders, preoperative sodium < 135 mEq/L, and longer operative times were found to be predictors of postoperative ICH requiring surgical evacuation (Table 4).

**Table 2:** Multivariable analysis comparing risk profiles for DVT occurring in-hospital (n = 69) versus post-discharge (n = 123) and PE occurring in-hospital (n = 29) versus post-discharge (n = 78)

Predictors	In-hospital DVT			Post-discharge DVT		
	OR	95% CI	p value	OR	95% CI	p value
Age per 10 years increase	1.28	1.06–1.55	**0.01**	1.28	1.12–1.47	< **0.001**
BMI per 5 kg/m^2^ increase	1.23	1.01–1.46	**0.03**	1.25	1.09–1.41	< **0.001**
Dependent functional status	2.63	1.18–5.27	**0.01**	0.82	0.34–1.68	0.62
Preoperative WBC > 12,000/µL	1.76	1.00–2.97	0.05	1.05	0.69–1.57	0.80
Steroid usage	1.38	0.71–2.52	0.32	2.17	1.39–3.30	< **0.001**
Admission to operation ≥ 2 days	1.85	1.08–3.19	**0.02**	2.02	1.37–2.98	< **0.001**
OR time per 60 min increase	1.26	1.13–1.39	< **0.001**	1.04	0.94–1.15	0.40

Predictors	In-hospital PE			Post-discharge PE		
	OR	95% CI	p value	OR	95% CI	p value
Age per 10 years increase	1.08	0.84–1.41	0.56	1.33	1.13–1.58	< **0.001**
Male gender	0.93	0.43–2.04	0.85	2.32	1.38–4.07	**0.002**
BMI per 5 kg/m^2^ increase	1.27	0.97–1.62	0.07	1.24	1.04–1.46	**0.01**
Dependent functional status	5.46	1.96–13.12	< **0.001**	2.15	0.94–4.31	0.05
OR time per 60 min increase	1.24	1.06–1.43	**0.004**	1.10	0.98–1.23	0.10

Bold p-values below 0.05 were considered as statistically significant

*BMI* body mass index, *DVT* deep venous thrombosis, *OR* operation room, *PE* pulmonary embolism, *WBC* white blood cell count

**Table 3:** Overview overall and specific risk factors for VTE

Overall risk factors		
Older age		
Higher BMI		

Specific risk factors		
	In-hospital	Post-discharge
DVT	Admission ≥ 2 days pre-op Longer operative times Dependent functional status	Admission ≥ 2 days pre-op Steroid usage
PE	Longer operative times Dependent functional status	Male gender

This study was underpowered to identify specific risk factors of in-hospital PEs

*BMI* body mass index, *DVT* deep venous thrombosis, *PE* pulmonary embolism, *pre-op* preoperatively

**Table 4:** Multivariable logistic regression analysis for ICH within 30 days after surgery (n = 72)

Predictor	Definition	ICH		
		OR	95% CI	p value
ASA classification	I–II	Ref	–	–
	III	1.45	0.74–3.11	0.31
	IV–V	3.23	1.50–7.59	**0.004**
Hypertension		2.27	1.38–3.75	**0.001**
Weight loss		4.42	1.48–10.67	**0.003**
Bleeding disorder		3.13	1.16–7.09	**0.01**
Preoperative SIRS		2.45	0.98–5.21	0.05
Preoperative sodium	135–145	Ref	–	–
	< 135	2.41	1.29–4.26	**0.004**
	> 145	1.14	0.06–5.59	0.90
Operative time	Minutes	1.20^a^	1.07–1.33	< **0.001**

Bold p-values below 0.05 were considered as statistically significant

*ASA* American Society of Anesthesiologists, *CI* confidence interval, *SIRS* systematic inflammatory response syndrome

^a^Inflated β-coefficients to odds ratio per 60 min increase

## Discussion

VTE is one of the most common major complications and reasons for readmission, and ICH the most common reason for reoperation among patients undergoing craniotomy for primary malignant brain tumors. This multicenter study provides novel and useful information regarding the timing of these events and identification of high-risk patients. The increased risk of VTE extends beyond the period of hospitalization, especially for PE, whereas ICH occurs predominantly within the first days after surgery. The VTE risk profile depends on the type of VTE (DVT vs. PE events) and the clinical setting (hospitalized vs. post-discharge patients).

The patient population in this study was technically classified as all those with primary malignant brain tumors based on ICD-9 codes. Gliomas represent close to 80% of primary malignant brain tumors [38], however, there is no standard ICD-9 code specific for glioma. The Central Brain Tumor Registry of the United States (CBTRUS) argues that multiple combinations of ICD histology codes can be used to define gliomas, and their approach was modeled in this study [38]. Therefore, these results are primarily applicable to glioma patients and should be put in the context of previous outcome research on glioma patients.

### Previous work

Several multicenter studies have previously investigated the short-term incidence of and risk factors for VTE after brain tumor surgery [3, 13, 39–45], of which four studies focused on glioma patients [3, 13, 39, 41]. From these studies, the rate of VTE following craniotomy is cited as 3.3–7.5% for glioma patients [3, 13, 39, 41] and 2.3–4.0% for brain tumors patients in general [11, 40–42, 44], with a follow-up ranging from solely the initial hospital stay to 6 weeks after surgery. The 30-day VTE rate was as high as 9.3% when asymptomatic DVTs were included too [3]. These results are comparable to the VTE rates found in the current study and suggest a higher rate of VTE in glioma patients postoperatively compared to other brain tumors.

Simanek et al. assessed the cumulative incidence of VTE over time after craniotomy for gliomas, demonstrating a major increase in the number of events in the first 3 months after surgery; however, no granular insight into the distribution of events within the first few weeks postoperatively was provided due to a low sample size. Neither did this study stratify for VTE type or the clinical setting of the patient [4].

Risk factors identified for VTE after craniotomy for gliomas are older age, history of craniotomy, history of VTE, coagulopathy, seizures, increased stay on the intensive care unit, prolonged hospital stay, residual tumor tissue, and absence of thromboprophylactic therapy [3, 4, 6, 12–15]. Missios et al. stratified for VTE type demonstrating different risk profiles for postoperative DVT and PE. Male gender, Hispanic ethnicity, and medium bed size were predictive for PE, whereas chronic heart failure was predictive for DVT [3]. Other predictors of postoperative VTE identified in the broader group of brain tumor patients were higher BMI, hypertension, functional dependence, lower Karnofsky Performance Scale (KPS) score, motor deficits, ventilator dependence, steroid usage, preoperative sepsis, longer operative times, and higher World Health Organization (WHO) tumor grade [11, 40, 42, 43, 46].

Prophylactic anticoagulation is a commonly used strategy to prevent VTE but should be carefully balanced against the risk of ICH. In previous studies, the rates of ICH following craniotomy for brain tumors is cited as 1.0–4.0% with a follow-up ranging between the initial hospital stay and long-term survival after surgery [6, 13–15, 44, 46–48]. However, definitions for major ICH varied between volumetric measurement of the hematoma, presence of symptoms, decrease in hemoglobin, or need for surgical evacuation of hematoma [14, 15, 21, 23, 24, 49].

Mantia et al. assessed the cumulative incidence of ICH over time after craniotomy for glioma. However, no time-to-event analysis was provided for the direct postoperative period due to a low sample size [23]. Neither did this study stratify for the clinical setting of the patient. Risk factors associated with ICH were history of craniotomy, use of bevacizumab, and therapeutic anticoagulation for VTE [13, 20–24]. The association between thromboprophylactic anticoagulation and ICH remains to be elucidated [15].

To our knowledge, the current study is the first large multicenter assessment including a descriptive time-to-event analysis for both VTE and ICH within 30 days after craniotomy for primary malignant brain tumors. Additionally, it is the first study that uses the NSQIP database to identify predictors of ICH after brain tumor resection. By addressing thrombotic outcomes as well as hemorrhagic outcomes, this study provides a meaningful direction for future research on thromboprophylactic treatment strategies. Lastly, the large sample size allows a stratification of both the descriptive and inferential analysis, demonstrating differences in risk profile and incidence over time based on VTE type (DVT vs. PE) and clinical setting (hospitalized vs. post-discharge patients).

### Limitations

Complication rates found in the current study can be conservative estimates if events were not reported back to the hospitals. VTEs were only coded as events if they were diagnosed and treated, thereby missing asymptomatic and undetected VTEs. The database additionally lacks several demographic variables identified in other studies as predictors. Tumor specific information (histology, size, location, residual tumor volume) and complication specific information (location and classification of DVT, PE, and ICH) was not available. However, both VTE and ICH were defined in the NSQIP database as complications requiring medical and surgical treatment, respectively, resulting in selection of the most clinically relevant events. Perhaps most importantly, no data is available regarding anticoagulation status and non-pharmaceutical prophylactic methods. Therefore, this study offers limited insight in the efficacy of different thromboprophylactic treatment strategies and their association with the occurrence of ICH. Selection bias can be introduced since institutions can selectively contribute patients to the NSQIP registry. There was a lower number of events due to separate analyses based on VTE type and clinical setting; however, our study was not underpowered for most outcome measures according to rule of 10 events per variable in the multivariable analysis [50]. Lastly, VTE and ICH events after the 30-day time period established in NSQIP are not accounted for in this study, although studies have demonstrated that the risk of VTE events remains non-negligible beyond 30 days postoperatively with incidences up to 26% in the first 12 months postoperatively [4–6]. Despite these limitations, this study provides useful insight into the rates, timing, and predictors of DVT, PE, and ICH after craniotomy for primary malignant brain tumors. Due to the multicenter nature of the NSQIP dataset, the results of this study may be more representative of typical management at all hospitals, including but not limited to tertiary care academic centers.

### Implications

The significant prevalence of VTE and ICH following craniotomy for primary malignant brain tumors found in the current study indicates that there is still room for improvement when it comes to monitoring and preventing these events. Rolston et al. demonstrated that the prevalence of VTE following a neurosurgical procedure registered in NSQIP has remained consistent over the last years [51]. This suggests that perioperative management still hasn’t improved effectively with regards to preventing VTE, despite the attention it receives in neurosurgical literature.

These results particularly encourage the need for continued awareness for VTE post-discharge, especially for PE, which has more lethal consequences. These PEs can also be considered more sudden since they were less often preceded by a known DVT. PEs preceded by a DVT, however, suggest inadequate treatment for the initial VTE event. It is possible that DVTs are less frequent post-discharge. It is our primary suspicion, however, that DVTs are underdetected after leaving the hospital because they are less frequently symptomatic and cannot be effectively screened for. It is also possible that patients develop symptomatic DVTs but are unaware of the signs and symptoms until they progress to PE, implicating a possible role for improved patient education in preventing morbidity caused by DVT and PE. In prospective randomized control trials investigating different VTE prophylaxis modalities, Goldhaber et al. screened all craniotomy patients prior to discharge and found 9.3% of patients to have VTE, most of which were asymptomatic in both studies [45].

Most guidelines recommend that prophylactic use of low-molecular weight heparin or unfractionated heparin should be considered in all cancer patients undergoing major surgery [16–19]. In patients undergoing operations for brain tumors, however, the benefits of anticoagulation should be carefully balanced against the risk of ICH [52, 53]. Although most guidelines support the use of pharmacological prophylaxis in patients with brain tumors, proper timing of prophylaxis remains controversial and the use of anticoagulation often depends on the surgeon’s preference [52–54]. Recommendations vary between administration throughout hospitalization [19], up to 7–10 days after surgery [16, 17, 55], until the patient is mobile [52], or timing based on the individual risk profile [56]. A lack of scientific evidence is primarily the cause of this variation in recommendations. Recent systematic reviews and meta-analyses of VTE prophylaxis in patients undergoing craniotomy for brain tumors have been performed [42, 57–60]. These analyses have compared different VTE prophylaxis modalities, as well as their safety and cost effectiveness, but they do not thoroughly investigate the efficacy of prophylaxis over time to determine a recommended duration. Only one clinical trial studied the effect of continued prophylaxis up to 12 months after surgery [15]. No significant association was found between prolonged prophylaxis and the rate of both VTE and ICH; however, the trial was stopped early because of expiration of study medication, and the control group received placebo instead of short-term prophylaxis. Many patients may not need or benefit from continuing thromboprophylactic therapy beyond discharge. Algattas et al. reviewed the safety and effectiveness of thromboprophylactic strategies and indicated that different regimens may have different efficacies depending on the patient’s VTE risk profile [57]. This highlights the importance of using the appropriate risk profile for optimizing postoperative management.

Since the NSQIP data does not contain information on thromboprophylactic strategies, the current study provides limited insight into the efficacy or safety of prophylactic anticoagulation and insufficient evidence to change the current clinical practice of thromboprophylaxis in patients undergoing operations for primary malignant brain tumors. Therefore, we concur with the current guidelines that recommend pharmaceutic prophylaxis (low-molecular weight heparin or unfractionated heparin) in combination with mechanical prophylaxis (anti-embolism stockings or intermittent pneumatic compression devices) postoperatively until the end of hospitalization or until the patient is mobile. Absolute contra-indications for these include recent ICH or another active major bleeding [16–19, 52, 53, 55, 56].

### Suggestions for future research

Despite its limitations, this study provides useful insight into the prevalence, timing, and risk factors of postoperative VTE and ICH after craniotomy for primary malignant brain tumors. The results of the current study demonstrate that there is still room for improvement, especially with regard to the prevention of PE after hospitalization. The distinct critical time periods for both thrombotic and hemorrhagic events suggest a potentially safe and effective role for continuing prophylactic anticoagulation post-discharge in high-risk patients. Additionally, the typical patient at risk for developing a VTE during hospitalization is not the same as the typical patient at risk for developing a VTE post-discharge. This is crucial for tailoring post-discharge management to the risk profile of the individual patient and suggests an important direction for future research. Therefore, future research should study the effects of timing of thromboprophylactic therapy, screening for asymptomatic events, and the effects of patient education on the occurrence of VTE and/or ICH. Additionally, future studies should construct prediction models for DVT, PE, and ICH and examine the effectiveness of tailoring postoperative thromboprophylaxis to the individual risk profile of patients undergoing craniotomy for primary malignant brain tumors.

## Conclusion

The increased risk of VTE experienced by patients with primary malignant brain tumors extends beyond the period of hospitalization, especially for PE, whereas ICH occurs predominantly in the first few days after surgery. The risk profile for VTE depends on the type of VTE and the clinical setting of the patient. VTE can have fatal consequences if not recognized early, therefore clinicians should have high suspicion during the postoperative period and a low threshold for specific monitoring and prevention.
